# Evaluation of Presacral Vascular Anatomy Using Contrast-Enhanced 3D-CT for Surgical Planning in Endoscopic Sacrocolpopexy

**DOI:** 10.3390/diagnostics16091385

**Published:** 2026-05-02

**Authors:** Akiko Abe, Yasushi Kotani, Chiharu Wada, Takaya Sakamoto, Yoko Kashima, Kosuke Murakami, Hisamitsu Takaya, Noriomi Matsumura

**Affiliations:** Department of Obstetrics and Gynecology, Faculty of Medicine, Kindai University, Sakai 590-0197, Japan

**Keywords:** laparoscopic sacrocolpopexy, robotic-assisted sacrocolpopexy, contrast-enhanced CT

## Abstract

**Background**: Endoscopic sacrocolpopexy (ESC) is a widely performed procedure for pelvic organ prolapse, with laparoscopic sacrocolpopexy (LSC) and robotic-assisted sacrocolpopexy (RSC) approaches. However, suturing to the anterior longitudinal ligament at the sacral promontory carries a risk of massive hemorrhage due to presacral vascular injury. This study aimed to determine the frequency of presacral venous variations considered clinically relevant during suturing at the promontory and to explore their association with perioperative outcomes using contrast-enhanced three-dimensional computed tomography (3D-CT). **Methods**: Among 319 consecutive ESC cases performed between 2014 and 2025, 265 patients who underwent preoperative contrast-enhanced CT were retrospectively analyzed in this single-center cohort study. Two vascular findings were defined as clinically significant: (1) anomalous drainage of the internal iliac vein into the contralateral common iliac vein and (2) a clearly visualized median sacral vein on 3D reconstruction. The clinical impact of vascular abnormalities was evaluated using surgical time, blood loss, and perioperative complication rates as indicators. Student’s *t*-test was used for comparing continuous variables, and the chi-squared test was used for comparing categorical variables. The data for this study were retrospectively collected from electronic medical records, anonymized, and then analyzed. **Results**: Anomalous internal iliac vein drainage was observed in 11.3% (30/265), and a visible median sacral vein was observed in 10.2% (27/265). Overall, 17.7% (47/265, CI: 13.2–22.2%) of patients had at least one clinically significant variation. There were no significant differences between the groups in terms of age, parity, BMI, operative time, blood loss, or perioperative complication rates. No cases required transfusion. **Conclusions**: Clinically significant presacral vein mutations were present in approximately 1 in 6 patients. The main findings of this study are that clinically significant presacral vascular mutations are relatively frequent (17.7%) in ESC and that there was no significant difference in perioperative outcomes between patients with and without vascular mutations. Clinically relevant presacral vascular variations are relatively common in ESC. Preoperative contrast-enhanced 3D-CT may support risk assessment and surgical planning.

## 1. Introduction

Pelvic organ prolapse (POP) is the descent of pelvic organs from their normal anatomical position, resulting in protrusion through the vagina. As the population ages, the number of affected patients has increased, and POP is recognized as a condition that significantly impairs women’s quality of life [1,2]. The relative lifetime risk of undergoing POP-related surgery is approximately 11% [3].

Traditionally, total vaginal hysterectomy (TVH), anterior and posterior colporrhaphy, and tension-free vaginal mesh (TVM) procedures have been used to treat POP. In recent years, laparoscopic sacrocolpopexy (LSC) and robotic-assisted sacrocolpopexy (RSC), both of which use mesh, have been widely adopted worldwide owing to their favorable surgical outcomes and lower recurrence rates [4,5,6,7,8,9].

Although LSC and RSC are minimally invasive procedures, they are technically demanding. In particular, suturing the mesh to the anterior longitudinal ligament at the sacral promontory is one of the most critical steps [10,11]. Major vessels, including the common iliac vein, internal iliac vein, and median sacral vein, run in close proximity to the anterior sacrum, and injury to these vessels may result in uncontrollable massive hemorrhage [12,13]. Anatomical variations in the internal and common iliac veins have been reported in 10–20% of cases based on CT analyses [14,15]. Therefore, a thorough preoperative understanding of the vascular anatomy around the promontory is crucial for safely performing endoscopic sacrocolpopexy. However, previous reports have mainly focused on anatomical classification, and the clinical significance of these variations during endoscopic sacrocolpopexy has not been sufficiently investigated. Furthermore, there are limited reports on the role of individualized anatomical assessment using preoperative 3D-CT in surgical planning [16,17]. In addition to anatomical considerations, preoperative vascular assessment has been investigated using various imaging modalities [18,19]. Contrast-enhanced CT, particularly in the venous phase, is widely used to evaluate pelvic vascular anatomy; however, its ability to visualize small veins such as the median sacral vein may be limited depending on contrast timing and spatial resolution [20]. Magnetic resonance venography (MRV) has also been proposed as an alternative modality with advantages in soft tissue contrast and the absence of radiation exposure. However, MRV is not routinely used in surgical planning due to its limited availability, longer acquisition time, and lower spatial resolution compared to CT [18,20,21]. Three-dimensional (3D) reconstruction based on contrast-enhanced CT provides intuitive visualization of complex vascular anatomy and spatial relationships between vessels and surgical landmarks. Compared with conventional multiplanar reconstruction, 3D-CT facilitates a more comprehensive understanding of patient-specific anatomy, which may be particularly useful for surgical planning in procedures such as endoscopic sacrocolpopexy. For these reasons, we focused on contrast-enhanced 3D-CT as a practical imaging modality for preoperative vascular assessment in this study.

We previously reported on the use of preoperative contrast-enhanced CT and three-dimensional reconstructed images (contrast-enhanced 3D-CT) to evaluate presacral vascular anatomy and avoid massive bleeding [22]. Based on this approach, we developed a system for discussing surgical strategies in preoperative conferences. Since then, we have accumulated additional cases with contrast-enhanced CT. The present study aimed to clarify the frequency of presacral vascular variations identified by preoperative contrast-enhanced 3D-CT in patients undergoing endoscopic sacrocolpopexy at our institution. We also compared perioperative outcomes according to the presence or absence of vascular variations and evaluated the impact of the 3D-CT assessment on the surgical strategy.

## 2. Materials and Methods

**Figure 1:**
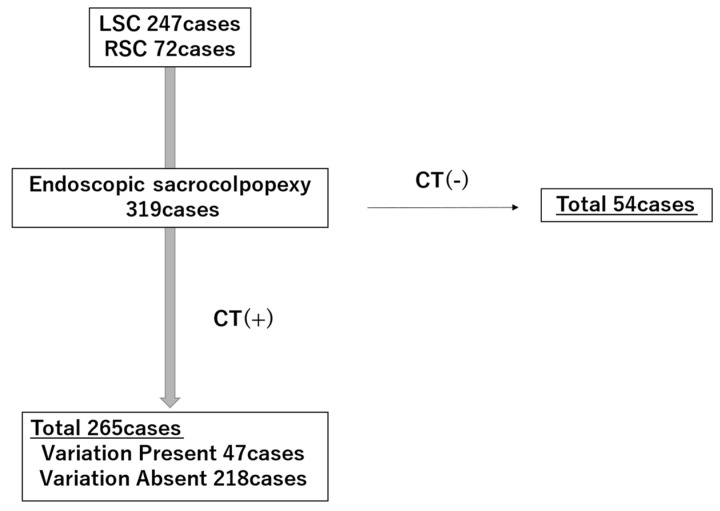
Flowchart of patient selection and classification based on contrast-enhanced CT findings.

From January 2014 to December 2025, a total of 319 LSC and RSC procedures were performed in the Department of Obstetrics and Gynecology, Kindai University Faculty of Medicine. Of these, 265 cases for which preoperative contrast-enhanced CT was performed were included in the analysis. We retrospectively evaluated presacral vascular anatomy using contrast-enhanced, 3D-reconstructed images. This study was conducted as a retrospective cohort study at a single institution. The patient selection process is illustrated in Figure 1. In addition, a comparison between included and excluded patients is provided in Supplementary Table S1.

The following two findings were defined as “clinically significant vascular variations” in this study: (1) an anomalous course in which one internal iliac vein drains into the contralateral common iliac vein, and (2) a clearly identifiable median sacral vein on 3D reconstructed images.

Cases with either of these findings were classified as variation-positive, while others were classified as variation-negative. This determination was made at a preoperative joint conference between gynecologists and radiologists. Evaluation of vascular anatomy on contrast-enhanced 3D-CT images was performed by experienced gynecologic surgeons in collaboration with radiologists during preoperative conferences. The presence or absence of vascular variations was determined based on consensus review. In cases of disagreement, a final decision was made through discussion. Although a standardized image interpretation protocol and independent blinded review were not employed, this consensus-based approach was intended to reflect real-world clinical decision-making.

We compared patient characteristics (age, parity, and BMI) and surgical outcomes (operative time, blood loss, and perioperative complication rate) between the groups. Student’s *t*-test was used for continuous variables, and a chi-square test was used for categorical variables. A *p*-value of less than 0.05 was considered statistically significant. The data for this study were retrospectively collected from electronic medical records, anonymized, and then analyzed.

Since 2018, our institution has routinely performed preoperative contrast-enhanced CT scans for all patients, in principle. Most patients who did not undergo CT were in the early stages of implementation or were unable to undergo imaging due to a contrast allergy. This study was approved by the Institutional Review Board of the Kindai University Faculty of Medicine (approval no. R06-185). Due to the retrospective nature of the study using anonymized clinical and imaging data, the requirement for written informed consent was waived, and an opt-out consent procedure was implemented in accordance with institutional guidelines. Information about the study was disclosed on the institutional website, and patients were given the opportunity to decline participation.

### 2.1. Indications for Laparoscopic Sacrocolpopexy

The indications for both LSC and RSC were defined as all cases of pelvic organ prolapse with a DeLancey Level I support defect. However, cases involving poor performance status or poorly controlled medical conditions, such as diabetes, were excluded. The choice between LSC and RSC was not based on predefined clinical criteria but was primarily determined by the availability of the robotic system and operating room scheduling.

### 2.2. Surgical Procedure

Endoscopic sacrocolpopexy (LSC or RSC) was performed according to standardized techniques at our institution. After establishing pneumoperitoneum, the sigmoid colon was retracted to expose the sacral promontory, and the peritoneum overlying the promontory was incised. Careful dissection was performed to expose the anterior longitudinal ligament at the sacral promontory while minimizing unnecessary presacral tissue dissection. Subsequently, a suture was placed in the anterior longitudinal ligament at the sacral promontory, and mesh fixation was performed. The rectovaginal and vesicovaginal spaces were developed, and the mesh was attached to the vaginal wall in a standard fashion. After appropriate tensioning, the mesh was secured to the sacral promontory, and the peritoneum was closed to cover the mesh.

The procedural steps of RSC were generally similar to those of LSC, with robotic assistance used primarily for dissection and suturing.

In this study, particular attention was paid to the surgical step involving dissection and suturing at the sacral promontory, as this is the area where presacral vascular injury may occur. Preoperative contrast-enhanced 3D-CT findings were used to identify vascular anatomy in this region and to guide surgical planning and intraoperative decision-making.

### 2.3. Postoperative Management

Patients undergoing LSC or RSC followed the same protocol. They began ambulating and taking oral fluids the day after surgery. Their urinary catheter was removed on postoperative day 2, and they were discharged on postoperative day 4. Patients were then monitored regularly in the outpatient clinic to confirm the absence of recurrence.

### 2.4. CT Acquisition and Reconstruction

All examinations were performed using a multidetector CT scanner (≥64-detector rows). Images were reconstructed with a slice thickness of 0.625 mm using a standard soft-tissue reconstruction kernel. A biphasic contrast protocol was used. The first phase was initiated 12 s after visual confirmation of contrast arrival at the monitoring region (hepatic–duodenal area), which corresponds to a late arterial–early venous phase. The second phase was acquired 80 s after the start of contrast injection, corresponding to the portal venous phase. This protocol was selected to enhance visualization of pelvic veins while maintaining sufficient arterial contrast, aiming to optimize the identification of presacral vascular anatomy for surgical planning. Iodinated contrast medium (iodine dose: 600 mgI/kg) was administered over 30 s. The concentration of contrast medium ranged from 300 to 370 mgI/mL, depending on availability. Although this protocol was designed for clinical applicability rather than strict radiological standardization, it enabled consistent visualization of major pelvic venous structures. However, the depiction of small-caliber veins may be affected by contrast timing, image quality, and individual hemodynamics.

### 2.5. 3D Reconstruction and Post-Processing

Three-dimensional (3D) reconstructions were generated from DICOM datasets using OsiriX MD version 14.0 (Pixmeo, Bernex, Switzerland). Vascular visualization was primarily performed using volume-rendering (VR) techniques. Initial segmentation was based on semi-automated thresholding to enhance contrast-filled vascular structures, followed by manual adjustment of window/level settings. To improve visualization of presacral veins, the surrounding soft tissue was selectively suppressed, and vessels were highlighted by adjusting opacity and color mapping. Minor manual editing, including cropping and removal of non-relevant structures, was performed when necessary to optimize visualization. All reconstructions were performed by operators familiar with pelvic anatomy and imaging interpretation, and the images were subsequently reviewed in multidisciplinary conferences for confirmation. This approach was intended to reflect practical clinical usage rather than a fully standardized segmentation protocol.

## 3. Results

**Figure 2:**
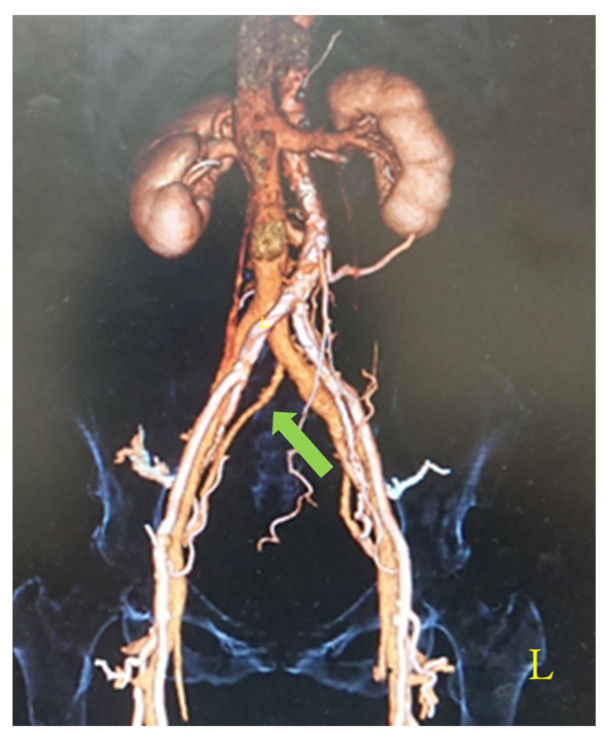
Representative contrast-enhanced CT image showing anomalous drainage of the unilateral internal iliac vein into the contralateral common iliac vein. Arrows indicate the anomalous venous course. This image represents a typical case observed in this study.

**Figure 3:**
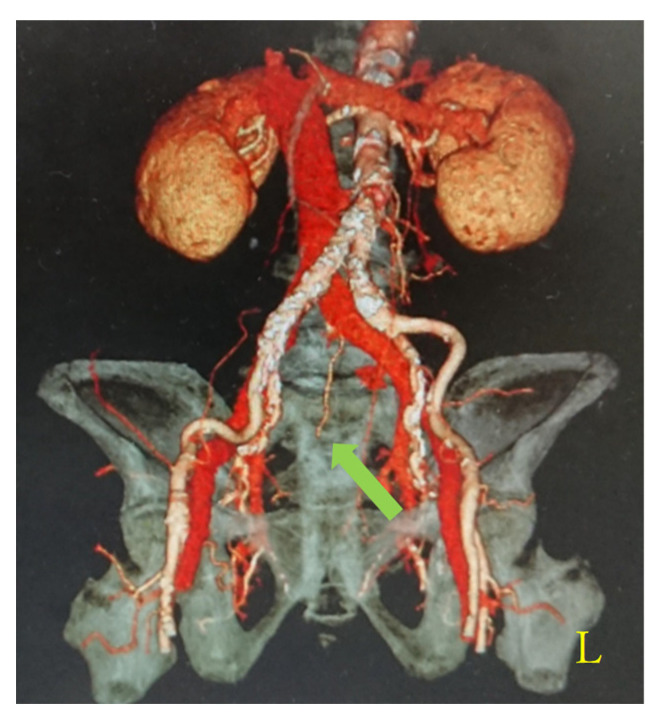
Representative contrast-enhanced CT image demonstrating the presence of a median sacral vein. Arrows indicate the median sacral vein. This image represents a typical case observed in this study.

Of the 265 cases that underwent contrast-enhanced CT, 30 cases (11.3%, 95% CI: 7.5–15.1%) demonstrated a course anomaly in which the unilateral internal iliac vein drained into the contralateral common iliac vein (Figure 2). The median sacral vein was identified on contrast-enhanced CT in an additional 27 cases (10.2%, 95% CI: 6.6–13.8%) (Figure 3). These two types of venous anomalies were present concurrently in eight cases. Overall, 47 cases (17.7%, 95% CI: 13.2–22.2%) were classified as having clinically relevant vascular variations.

Among the patients who underwent contrast-enhanced CT, 47 were assigned to the anomaly group and 218 to the no-anomaly group. Patient characteristics and surgical outcomes were compared between the two groups (Table 1). There were no significant differences in baseline characteristics, including age, parity, and body mass index. Similarly, no statistically significant differences were observed in operative time (221 vs. 224 min), intraoperative blood loss (27 vs. 45 mL), or perioperative complication rates (2.4% vs. 4.1%) between the groups. Intraoperative blood loss was summarized as mean ± standard deviation and median [interquartile range], given its skewed distribution (27.3 ± 41.0 vs. 44.6 ± 80.2 mL; median [IQR]: 10 [10–50] vs. 10 [10–20] mL).

Importantly, no cases of massive hemorrhage requiring blood transfusion were observed in either group. Furthermore, no cases of vascular injury or significant bleeding related to dissection or suturing at the sacral promontory were observed in this cohort.

The proportion of surgical approaches differed significantly between the CT and non-CT groups, with a higher proportion of LSC in the non-CT group (92.6% vs. 74.3%, *p* < 0.01) (Supplementary Table S1).

## 4. Discussion

The main findings of this study are as follows: First, clinically relevant presacral vascular variations were identified in 17.7% of patients undergoing endoscopic sacrocolpopexy. Second, no significant differences in perioperative outcomes were observed between patients with and without vascular variations. Therefore, comparisons of perioperative outcomes between groups should be interpreted with caution, given the potential influence of confounding factors and the retrospective nature of the study. Third, preoperative evaluation using contrast-enhanced 3D-CT enabled visualization of individual vascular anatomy and may contribute to surgical planning. Importantly, no bleeding events directly related to sacral promontory dissection or suturing were observed in this study, which may reflect the effectiveness of preoperative anatomical assessment and careful surgical technique.

In recent years, the importance of preoperative imaging for surgical planning has been increasingly recognized in complex endoscopic procedures. Advances in three-dimensional image reconstruction have made it possible to evaluate individual anatomical features in detail before surgery. In the context of pelvic organ prolapse surgery, where endoscopic sacrocolpopexy has become widely adopted, careful preoperative preparation is essential for safely performing this technically demanding procedure.

In particular, the vascular anatomy of the anterior sacrum shows considerable interindividual variability and requires careful attention [23]. During suturing to the anterior longitudinal ligament at the sacral promontory, the presence of vascular variations may increase the risk of vascular injury and massive hemorrhage [22,24,25,26]. If vascular anomalies are not identified preoperatively, surgeons may need to extend the dissection intraoperatively to identify vascular structures. However, excessive dissection itself has been reported to increase the risk of bleeding from the presacral venous plexus [13].

**Figure 4:**
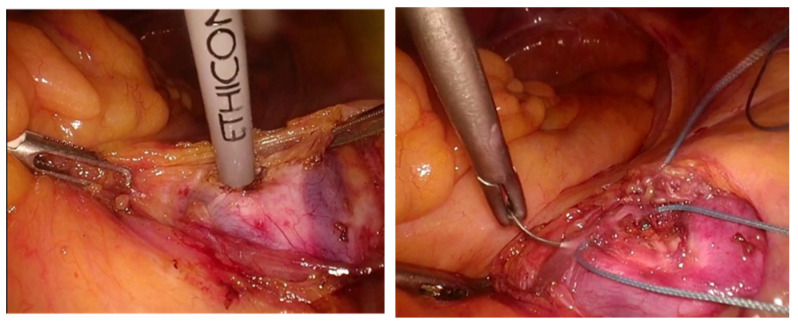
Illustrative example showing how preoperative contrast-enhanced 3D-CT findings may aid in identifying presacral vascular anatomy during surgery. This figure is presented for explanatory purposes and does not represent a systematic correlation between imaging findings and surgical difficulty or outcomes.

Preoperative visualization of vascular anatomy using contrast-enhanced 3D-CT allows surgeons to recognize vascular variations before surgery. This may help surgeons anticipate anatomical challenges and potentially avoid unexpected vascular injury during dissection and suturing at the sacral promontory (Figure 4). Figure 4 provides an illustrative example of anatomical correlation in a case with anomalous drainage of the right internal iliac vein into the left common iliac vein, demonstrating both the preoperative imaging findings and the corresponding intraoperative laparoscopic view during suturing at the sacral promontory; however, this study did not systematically evaluate the relationship between imaging findings and surgical difficulty or bleeding risk.

**Figure 5:**
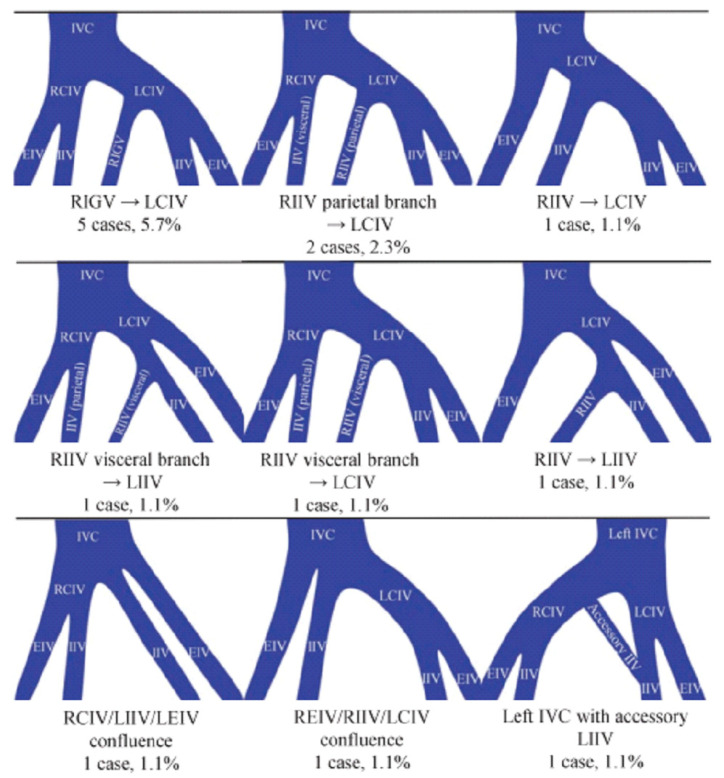
Classification and frequency of anatomical abnormalities of presacral vessels based on the report by Kato et al. [27]. Abbreviations: RIGV: Right inferior gluteal vein, LCIV: Left common iliac vein, RIIV: Right internal iliac vein, LIIV: Left internal iliac vein, RCIV: Right common iliac vein, LEIV: Left external iliac vein, REIV: Right external iliac vein, IVC: Inferior vena cava, EIV: External iliac vein; IIV: Internal iliac vein.

Previous studies have reported the prevalence of vascular variations using CT; however, the application of contrast-enhanced CT for surgical planning remains limited [22]. Albanesi et al. demonstrated the feasibility of 3D modeling using contrast-enhanced CT but did not evaluate the prevalence of vascular anomalies. In contrast, Kato et al. reported 9 patterns of vascular variation using plain CT, with an incidence of 15.9% [27]. These nine patterns are illustrated in Figure 5. In the present study, we focused on two types of vascular variations that are considered to be particularly relevant during endoscopic sacrocolpopexy: (1) anomalous drainage of the unilateral internal iliac vein into the contralateral iliac vein and (2) the presence of a median sacral vein. The first type of variation corresponds to the right upper and middle right patterns in Figure 5 of Kato et al., which represent internal iliac vein drainage into the contralateral common iliac vein or internal iliac vein. In this study, these patterns were collectively evaluated as a single category. The second variation, defined as the presence of a median sacral vein, refers to a vein located along the midline that is clearly visualized on CT imaging. This corresponds to four categories in Kato et al.’s classification (upper left, upper middle, middle left, and middle middle in Figure 5). Although Kato et al. described these as branches of the inferior gluteal vein or internal iliac vein, we considered that precise subclassification of these small venous branches may be limited by imaging resolution. Therefore, we grouped these findings together and defined them as the presence of a median sacral vein. In contrast, the patterns shown in the lower left and lower middle of Figure 5 were reported by Kato et al. to have limited clinical relevance and were therefore excluded from the present analysis. In addition, no cases corresponding to the lower right pattern were identified in our cohort. Thus, rather than adopting a detailed anatomical classification, we deliberately focused on two types of vascular variations that are likely to be clinically relevant during endoscopic sacrocolpopexy.

Based on these considerations, we deliberately focused on clinically relevant variations rather than adopting a detailed classification system. As a result, vascular variations were identified in 17.7% of cases, indicating that surgeons may encounter such variations in approximately one out of every five to six patients.

Although contrast-enhanced CT has several disadvantages, including contrast allergy, radiation exposure, and increased cost [28,29,30], preoperative identification of vascular anatomy allows for improved risk awareness and information sharing among the surgical team [31,32,33,34]. This facilitates multidisciplinary collaboration, including appropriate surgeon allocation during critical steps and anesthetic risk management. At our institution, vascular anatomy is routinely reviewed preoperatively in multidisciplinary conferences.

This study has several limitations. First, this was a retrospective study conducted at a single institution, which may introduce selection bias. In particular, contrast-enhanced CT was not performed in all patients during the early study period, and the non-CT group mainly consisted of cases from the initial phase or those with contraindications, potentially affecting the comparability of the groups. In addition, the selection of LSC or RSC was not standardized and was influenced by logistical factors such as robotic system availability. This may have introduced heterogeneity that could affect operative time, blood loss, and complication rates. Second, the definition of “clinically relevant vascular variation” in this study was based on two selected patterns considered to be important for surgical procedures. Although this approach was intended to focus on clinically meaningful variations, it may not fully capture the entire spectrum of anatomical variations. Third, small vessels, such as the median sacral vein, may not always be visualized on CT, and additional vascular structures may be identified intraoperatively. Fourth, the overall incidence of massive bleeding was very low in this cohort, which limits the ability to evaluate the direct impact of preoperative CT on bleeding-related outcomes. In addition, only univariate analyses were performed in this study, and potential confounding factors such as age, BMI, and surgical approach were not adjusted for. Therefore, the results of perioperative outcome comparisons should be interpreted with caution. The CT acquisition protocol used in this study was optimized for clinical surgical planning rather than standardized radiological vascular imaging, which may influence the visualization of small-caliber veins. Fifth, the assessment of vascular variations was based on consensus interpretation rather than independent blinded review, and interobserver variability was not formally evaluated, which may affect reproducibility. The lack of a standardized interpretation protocol may introduce potential bias and affect the objectivity and reproducibility of the findings. Finally, this study does not demonstrate that preoperative contrast-enhanced CT directly reduces intraoperative complications. Future multicenter prospective studies are required to further clarify the clinical impact of preoperative imaging on surgical outcomes.

## 5. Conclusions

This study demonstrated that clinically relevant presacral vascular variations are relatively common in patients undergoing endoscopic sacrocolpopexy. Preoperative evaluation using contrast-enhanced 3D-CT may be useful for anatomical risk assessment and surgical planning by enabling visualization of vascular anatomy. However, its impact on perioperative outcomes remains unclear and should be evaluated in future prospective studies.

## Figures and Tables

**Table 1:** Comparison of patient characteristics and perioperative outcomes according to the presence of vascular variation.

	Variation Present *n* = 47	Variation Absent *n* = 218	*p* Value
Age (years)	69.6 ± 7.7	70.1 ± 8.0	0.72
Parity	2.2 ± 0.8	2.3 ± 0.7	0.81
BMI	23.7 ± 2.8	24.4 ± 3.3	0.17
Operative time (min)	221.3 ± 44.8	224.1 ± 52.5	0.73
Blood loss (mL)	27.3 ± 41.0	44.6 ± 80.2	0.15
Perioperative Complications (%)	2.4 (1/47) Intestinal obstruction 1	4.1 (9/218) Intestinal injury 2 Severe subcutaneous emphysema 2 Abdominal wall incisional hernia 2 Bladder injury 1 Ileus 1 Pelvic peritonitis 1	0.51

## Data Availability

The data presented in this study are available on request from the corresponding author due to the data are not publicly available due to privacy or ethical re-strictions.

## Supplementary Materials

The following supporting information can be downloaded at: https://www.mdpi.com/article/10.3390/diagnostics16091385/s1, Table S1: Comparison of baseline characteristics between patients with and without contrast-enhanced CT.

## Abbreviations

The following abbreviations are used in this manuscript:

LSC	Laparoscopic sacrocolpopexy
RSC	Robotic-assisted sacrocolpopexy
3D-CT	Three-dimensional computed tomography
POP	Pelvic organ prolapse
