# Supplementary material for: Evaluation of Presacral Vascular Anatomy Using Contrast-Enhanced 3D-CT for Surgical Planning in Endoscopic Sacrocolpopexy
Source: Diagnostics (Basel). 2026 May 2;16(9):1385. doi: 10.3390/diagnostics16091385 (PMC13163452; doi:10.3390/diagnostics16091385)
Supplement: Supplementary file 1 [file diagnostics-16-01385-s001.zip › diagnostics-4203935-supplementary.pdf]

Supplementary Table S1. Comparison of baseline characteristics between patients with and without contrast-enhanced CT

	CT (+)<br>n=265	CT (-)<br>n=54	P value
Age (years)	70.0±7.9	67.9±9.0	0.08
Parity	2.3±0.7	2.3±0.6	0.58
BMI	24.3±3.2	24.3±3.3	0.99
LSC, n (%)	197 (74.3%)	50 (92.6%)	<0.01
RSC, n (%)	68 (25.7%)	4 (7.4%)	
